# Prevalence and factors associated with *Babesia bigemina* infection in Crioula Lageana cattle breed

**DOI:** 10.1590/S1984-29612025018

**Published:** 2025-04-07

**Authors:** Mariana da Silva Casa, Julio de Mattos Vettori, Ketriane Mota de Souza, Paulo Ricardo Benetti Todeschini, Luiz Cláudio Miletti, Carla Ivane Ganz Vogel, André Luís Ferreira Lima, Joandes Henrique Fonteque

**Affiliations:** 1 Programa de Pós-graduação em Ciência Animal, Universidade do Estado de Santa Catarina – UDESC, Lages, SC, Brasil; 2 Departmento de Medicina Veterinária, Universidade do Estado de Santa Catarina – UDESC, Lages, SC, Brasil; 3 Programa de Pós-graduação em Bioquímica e Biologia Molecular, Universidade do Estado de Santa Catarina – UESC, Lages, SC, Brasil; 4 Departmento de Produção Animal e Alimentos, Universidade do Estado de Santa Catarina – UDESC, Lages, SC, Brasil; 5 Departamento de Zootecnia e Desenvolvimento Rural, Universidade Federal de Santa Catarina – UFSC, Florianópolis, SC, Brasil

**Keywords:** Beef cattle, epidemiology, native breed, Bovino de corte, epidemiologia, raça nativa

## Abstract

The objective of this study was to determine the prevalence of *Babesia bigemina* infection and associated risk factors in Crioula Lageana cattle, a Brazilian native breed known for its tick resistance. Blood samples were collected from 311 registered cattle (62 males, 249 females) from conservation nucleus properties in Santa Catarina State, Brazil. Samples underwent DNA extraction and nested PCR targeting the rap-1a gene for *B. bigemina* detection. Animals were categorized by sex, age class, and tick presence during sampling. An epidemiological questionnaire assessed potential risk factors. The overall *B. bigemina* prevalence was 60% (186/311; 95% CI: 56,95%-62,67%). Males showed significantly higher infection rates (79%; 49/62) compared to females (55%; 137/249; OR=3.36, p<0.001). Bulls (81%; 26/32) and calves (78%; 56/72) exhibited higher infection rates than cows (50%; 70/141) and heifers (52%; 34/66; p<0.001). Tick presence during sampling increased infection probability (OR=2.00, p=0.006). Contact with other animal species (OR=1.57, p=0.037) and regular veterinary care (OR=6.77, p=0.009) were identified as significant risk factors. Results indicate enzootic instability in the studied population, with distinct sex-based susceptibility patterns. These findings provide baseline data for developing targeted control strategies for *B. bigemina* in Crioula Lageana breeding programs.

## Resumo

O objetivo deste estudo foi determinar a prevalência de infecção por *Babesia bigemina* e os fatores de risco associados em bovinos da raça Crioula Lageana, uma raça nativa brasileira, conhecida por sua resistência a carrapatos. Amostras de sangue foram coletadas de 311 bovinos registrados (62 machos e 249 fêmeas), provenientes de propriedades pertencentes a núcleos de conservação no estado de Santa Catarina, Brasil. As amostras foram submetidas à extração de DNA e à técnica de PCR aninhada, direcionada ao gene *rap-1a* para detecção de *B. bigemina*. Os animais foram categorizados por sexo, classe etária e presença de carrapatos no momento da coleta. Um questionário epidemiológico foi aplicado para avaliar potenciais fatores de risco. A prevalência geral de *B. bigemina* foi de 60% (186/311; IC 95%: 56,95%-62,67%). Os machos apresentaram taxas de infecção significativamente maiores (79%; 49/62) em comparação com as fêmeas (55%; 137/249; OR=3,36, p<0,001). Touros (81%; 26/32) e bezerros (78%; 56/72) exibiram maiores taxas de infecção do que vacas (50%; 70/141) e novilhas (52%; 34/66; p<0,001). A presença de carrapatos no momento da coleta aumentou a probabilidade de infecção (OR=2,00; p=0,006). O contato com outras espécies animais (OR=1,57; p=0,037) e o cuidado veterinário regular (OR=6,77; p=0,009) foram identificados como fatores de risco significativos. Os resultados indicam instabilidade enzoótica na população estudada, com padrões distintos de suscetibilidade baseados no sexo. Esses achados fornecem dados fundamentais para o desenvolvimento de estratégias de controle direcionadas para *B. bigemina* em programas de manejo e melhoramento genético da raça Crioula Lageana.

## Introduction

Babesiosis is a tick-borne disease of global importance, affecting cattle populations in tropical and subtropical regions (Figueroa et al., 1998) and resulting in considerable economic losses. In Brazil, *Babesia bigemina* is one of the primary causative agents of bovine babesiosis, transmitted by the tick *Rhipicephalus microplus* (Guglielmone, 1995). The clinical and subclinical infections can reduce productivity, increase treatment costs, and lead to trade restrictions (Bock et al., 2004).

Although taurine breeds are generally more susceptible to babesiosis, less is known about disease’s prevalence and epidemiological patterns in locally adapted cattle populations, such as the Crioula Lageana breed, native to the Santa Catarina Plateau. This breed has been recognized for its hardiness and adaptability (Cardoso et al., 2014), and understanding its infection profile could provide insights into genetic and management-related factors that influence disease occurrence, given how certain studies have associated variations in susceptibility with the breed of animal (Bock et al., 1997; 1999). Clarifying how intrinsic factors (such as breed, age and sex) and extrinsic factors (e.g., contact with other animal species, tick presence, and herd-level management practices) affect *B. bigemina* infection risk is crucial for developing targeted control strategies (Bilhassi et al., 2014).

Despite existing research on bovine babesiosis in southern Brazil, studies don’t usually focus on Crioula Lageana herds (Souza et al., 2002; Canever et al., 2014; Vieira et al., 2019). Such information is particularly relevant as these animals represent a valuable genetic resource and are raised in environments where babesiosis may be endemic but under-characterized and applying PCR analysis to diagnose *B. bigemina* infection enables the identification of healthy carrier animals and, thus, a more accurate determination of the prevalence (Oliveira-Sequeira et al., 2005; Buling et al., 2007).

The aim of this study was to present novel epidemiological data on the prevalence of *B. bigemina* infection in Crioula Lageana cattle using molecular techniques and determine the risk factors associated with the acquisition of the agent. By doing so, we hope to contribute to better disease control measures, preserve the genetic integrity of this native breed, and improve the overall productivity and sustainability of local cattle farming systems

## Materials and Methods

### Determination of sample size

The following formula (Thrusfield, 2007) was used to evaluate the prevalence of *B. bigemina* in the Crioula Lageana bovine population:


$${\text{n}}_{0}=\frac{{1,96}^{2}[\text{p}(1-\text{p})]}{{\left(\text{d}\right)}^{2}}$$ (1)

where ${\text{n}}_{0}$ is the number of samples, p is the expected prevalence, and d is the error margin. Assuming an estimated prevalence of 50% in the positive samples, a confidence interval of 95%, and a margin of error of 5%, the result was 384 animals. However, because it has a finite population, the following calculation was made:


$$\text{n}=\frac{Nx{\text{n}}_{0}}{\text{N}+{\text{n}}_{0}}$$ (2)

where *N* is the total number of animals in the population; in this case, it was 1500 animals. Based on this calculation, the final number of 306 animals to be sampled was achieved.

### Animals and obtaining samples

Blood samples were collected during the autumn of 2016 from 311 young and adult Crioula Lageana male and female cattle registered at the Brazilian Association of Breeders of the Crioula Lageana Breed (ABCCL), chosen randomly, coming from in situ conservation nucleus properties, located in the Santa Catarina Plateau.

The animals were separated into categories and samples, chosen randomly, were collected from 32 bulls (males >2 years), 141 cows (females >2 years), 66 heifers (females between 1 and 2 years), and 72 calves (males and females up to 1 year). The absence of steers was due to the fact that the samples were derived from in situ conservation core properties of the breed; therefore, non-breeding males are sold.

The animals were divided into 62 males and 249 females, regardless of age, for sex analysis. Vacuum collection tubes containing 10% EDTA acid were used as anticoagulants. The samples were frozen at −20 °C after collection until DNA extraction.

### Physical exam

Physical examination was performed to verify clinical signs compatible with clinical disease, including heart rate (HR), respiratory rate (RR), ruminal motility (RM), rectal temperature, and mucosal staining.

### DNA extraction

After thawing, the blood samples were immediately subjected to DNA extraction using a ReliaPrep Blood gDNA Miniprep System (PROMEGA) commercial kit, according to the manufacturer's instructions. After extraction, the concentration of each DNA sample was measured using a NanoDrop 2000 spectrophotometer (Thermo Scientific) and diluted to maintain a minimum concentration of 20 ng/μL.

### Molecular analysis

PCR and nested PCR (n-PCR) were used to amplify *B. bigemina* DNA. Primer sets used in these reactions have been described previously (Figueroa et al., 1993). All the DNA samples were subjected to both reactions. The PCR reaction was performed in 0.2 mL microtubes, where a final volume of 25 μL of a solution containing 1 U of enzyme Taq Polymerase GoTaq Hot Start Polymerase, (Promega) was added; 8.5 pmol of each primer (BiIA: 5ʹ-CAT CTA ATT TCT CTC CAT ACC CCT CC-3ʹ e BiIB: 5ʹ-CCT CGG CTT CAA CTC TGA TGC CAA AG-3ʹ), 0.2 mM nucleotide (dNTPs); 3.5 mM magnesium chloride; 5 μL of 5X Green GoTaq Flexi Buffer (Promega); 3 μL DNA (concentration between 20 and 100 ng/μL) and ultrapure water to adjust the final volume and reagent concentration. Positive and negative controls using the same parameters mentioned above and replacing only the use of genomic DNA with ultrapure DNase-free water were used for each reaction, the latter being used to ensure the quality and specificity of the technique. The same reaction mix was used for n-PCR, replacing only animal DNA with 2 μL of the first PCR product, and primers used by n-PCR-specific (BiIAN: 5ʹ-CGC AAG CCC AGC ACG CCC CGG TGC-3ʹ e BiIBN: 5ʹ-CCG ACC TGG ATA GGC TGT GTG ATG-3ʹ).

The temperature conditions applied to the thermocycler (Biocycler) for both reactions involved initial denaturation at 95 °C for two minutes, followed by 30 cycles of 94 °C for 1 minute, 54.2 °C for 1 minute and 73 °C for 1 minute, and a final extension at 73 °C for 7 minutes.

Electrophoresis of the amplification products was performed in a horizontal tray using a 2% agarose gel plus Unisafe Dye 20,000X (Uniscience). A 100 bp molecular weight marker was loaded in the first lane as a standard to determine the size of the sample bands. The conditions of the electric source were 140 Volts and 400 mA 60 minutes, and visualization was conducted by exposure to ultraviolet light. Bands close to 278 bp in size were considered positive for *B. bigemina* in the first reaction, and bands approximately 170 bp in size were considered positive in the second reaction.

### Associated factors

An epidemiological questionnaire containing questions related to the property profile was administered to the owners of each property analyzed to determine the factors associated with the development of babesiosis caused by *B. bigemina*.

### Statistical analysis

Univariate analysis was performed to compare *B. bigemina* infection rates according to sex, category, and tick presence at the time of sampling using the chi-square test (*p* ≤ 0.05) and odds ratio analysis. The statistical model applied to the questionnaire data consisted of a univariate analysis using the chi-square test (*p* < 0.05).

Multivariate analysis using logistic regression analysis (*p* < 0.05) was performed for questions with significant results in the first analysis. Questions that presented multicollinearity in the second analysis were excluded from the evaluation to check the association between the presence or absence of the agent and the associated factors. All analyses were performed using R-4.4.2 software.

## Results

### Prevalence

The prevalence of positive samples was 60% (186/311; 56,95%-62,67%) for *B. bigemina*. All positive animals were considered subclinical, as no change in the variables evaluated on physical examination was verified, and all remained within the reference values for the species.

By assessing the sex and category of animals in relation to the presence or absence of infection, we found that 249 (249/311; 80%) were female, and 137 (137/249; 55%) of the animals sampled were positive for the agent. Of the 62 males (62/311; 20%), 49 (49/62; 79%) tested positive. There was a significant difference (*p* < 0.001) in the rates of parasitized animals in the comparison between the sexes with males being 3.357 times more likely to be parasitized than females (OR=3.36).

Out of the 32 (32/311; 10%) bulls evaluated, 26 (26/32; 81%) were positive, whereas 34 (34/66; 52%) of the 66 (66/311; 21%) heifers presented the same result. Among the 141 (141/311; 45%) cows, 70 (70/141; 50%) had *B. bigemina*-compatible electrophoresis bands, whereas 56 (56/72; 78%) of the 72 (72/311; 23%) calves displayed these bands.

There was a significant difference (*p* = 0.009) between bulls and heifers, and bulls were 4.078 times more likely to be infected by *B. bigemina* than heifers. In another comparison, bulls were 4.3 times more likely to be infected than cows (*p* = 0.002). Among heifers and calves (*p* = 0.002), the chance of calves acquiring an infection was 3.2 times higher than that of heifers. Calves were 3.5 times more likely to develop infection than cows (*p* < 0.001).

The relationship between the presence or absence of ticks parasitizing animals at the time of sampling and the presence or absence of *B. bigemina* infection was also evaluated. This analysis showed a relationship (*p* = 0.006) between the variables in the chi-square test, with a 2.002-time greater chance of the animals in the presence of ectoparasites acquiring hemoprotozoans.

### Associated factors

In the analysis of the answers to the questionnaires administered to the owners, the productive purpose of the breeding, contact of the cattle with other animal species, veterinary assistance, the period of greatest tick infestation, the time of treatment for ticks and babesiosis, and the most parasitized categories differed in the univariate analysis of associated factors (Table 1).

**Table 1:** Univariate analysis of factors associated with *B. bigemina* infection in Crioula Lageana cattle.

**Variables**		***B. bigemina* infection**				***p***
		**Positive**		**Negative**		
		**N**	**%**	**N**	**%**	
**Number of animals on the property**						
51 to 100		31	10	16	5.2	0.440
100+		155	49.8	109	35	
**Productive Purpose of Creation**						
Meat, reproduction, and sale		88	28.3	77	24.8	<0.001
Meat		12	3.9	5	1.6	
Reproduction and sale		33	10.6	28	9	
Meat and sale		34	10.9	4	1.3	
Sale		19	6.1	11	3.5	
**Property size**						
50 to 100 ha		12	3.9	5	1.6	0.498
Greater than 100 ha		174	55.9	120	38.6	
**Contact of cattle with other animal species**						
Horse, dog, and sheep		40	12.9	32	10.3	0.002
Swine		19	6.1	11	3.5	
Horse and dog		34	10.9	4	1.3	
Horse, pig, dog, and cat		33	10.6	28	9	
Equine, dog, cat, bird, and sheep		48	15.4	45	14.5	
Equine, dog, cat and wild animals		12	3.9	5	1.6	
**Contact with cattle from other properties**						
Yes		107	34.4	64	20.6	0.325
No		79	25.4	61	19.4	
**Animal Replacement**						
Own herd		59	19	43	13.8	0.711
Own herd and other properties		127	40.8	82	26.4	
**Veterinary care**						
Yes		93	29.9	47	15.1	0.041
No		93	29.9	78	25.1	
**Previous Babesiosis Cases**						
Yes		74	23.8	36	11.6	0.062
No		112	36	89	28.6	
**Period of greatest tick infestation**						
Fall		45	14.5	33	10.6	0.001
Summer		67	21.5	56	18	
Fall and spring		34	10.9	4	1.3	
Summer and fall		40	12.9	32	10.3	
**Presence of hematophagous insects**						
Yes		153	49.2	97	31.2	0.385
No		33	10.6	28	9	
**Tick control**						
Yes		141	45.3	92	29.6	0.759
No		45	14.5	33	10.6	
**Acaricides Used**						
Pyrethroids		86	27.7	41	13.2	0.061
Organophosphates and avermectins		67	21.5	56	18	
Avermectines		33	10.6	28	9	
**Time of tick and babesiosis treatments**						
Fall		45	14.5	33	10.6	0.002
Spring and fall		34	10.9	4	1.3	
Spring, summer, and fall		48	15.4	45	14.5	
Summer		19	6.1	11	3.5	
Summer and fall		40	12.9	32	10.3	
**Most tick-parasitized categories**						
Pregnant, lactating cow, and calf		34	10.9	4	1.3	<0.001
Lactating cow		12	3.9	5	1.6	
Calf		140	45	116	37.3	

Questions with a *p*-value <0.05 were considered to have significant results in univariate analysis and thus submitted to multivariate analysis. Factors presenting multicollinearity were excluded from the model because the real relationships between the variables were not determined (Table 2).

**Table 2:** Multivariate analysis of factors associated with *B. bigemina* infection in Crioula Lageana cattle.

**Risk Factor**	***p***	**OR**	**95% CI**	**Coefficient**	**S.E**
Productive Purpose of Creation	0.259	1.074	0.949 - 1.217	0.072	0.064
Contact of cattle with other animal species*	0.037	1.570	1.027 - 2.401	0.451	0.217
Veterinary care*	0.009	6.770	1.596 - 28.715	1.912	0.737
Time of tick and babesiosis treatments	0.074	0.861	0.730 - 1.015	-0.150	0.084

* Significant association at 5% level. OR = Odds ratio, CI=95%, confidence interval, S.E. = standard error of estimation.

Multivariate analysis using binary logistic regression was used to verify whether these associated factors were predictors of *B. bigemina* infection. Analyzing the *p*-value of each predictor verified that the productive purpose of rearing and the time of treatment for ticks and babesiosis were not significant predictors of the model, whereas the other variables were considered significant.

## Discussion

This study offers the first detailed molecular epidemiological assessment of *Babesia bigemina* infection in the Crioula Lageana breed, a genetically valuable and locally adapted cattle population from the Santa Catarina Plateau in southern Brazil. (Fino et al., 2013). The findings indicate that these herds are in a state of enzootic instability for *B. bigemina*, a condition in which herd immunity and infection risk balance precariously below the threshold typically associated with stable endemicity (Guimarães et al., 2011). Despite its importance as a high-quality genetic source, few studies determined the health profile of this breed and its behavior in the face of various diseases (Cardoso et al., 2013, 2014; Fino et al., 2013).

The observed prevalence, approximately 60% using a PCR-based assay, aligns with ongoing parasite circulation but is markedly lower than the high seroprevalence rates previously reported in some southern Brazilian herds, specifically Paraná, with a 87.5% positivity rate using an ELISA serological test on cattle (Vidotto et al., 2006). This discrepancy underscores the importance of diagnostic methodology: serological tests detect antibodies and thus reflect cumulative, historical exposure, while PCR identifies active infections.

Taurine breeds are considered the most susceptible to ticks and babesiosis (Jonsson et al., 2008; Piper et al., 2010). However, the Crioula Lageana breed is resistant to ectoparasite infection (Cardoso et al., 2014) and future studies should aim to verify tolerance to babesiosis and other diseases. Although no clinical outbreaks or severe disease were noted by herd owners, the presence of ongoing infections suggests that subclinical parasite transmission persists. These results emphasize the need to remain vigilant, since enzootic instability heightens the potential for sudden and severe outbreaks if management conditions change or immunity wanes.

The herd in the Northern Plateau of Santa Catarina had a seroprevalence of 84.5% for *B. bigemina* using an indirect immunofluorescence test (Souza et al., 2002). More recently, an outbreak of babesiosis and anaplasmosis occurred in Ponte Alta, Santa Catarina State, where 63.6% of the animals tested positive for *B. bigemina* using the multiplex-PCR technique (Canever et al., 2014).

Another study evaluated the prevalence of Cattle Tick Fever agents and found that 16.73% of herds were positive for *B. bigemina* using the multiplex-PCR technique. These indices represent an enzootic instability situation for babesiosis in herds in the region, necessitating vector control (Vieira et al., 2019). The results of this study on the Crioula Lageana breed corroborated the findings for this region and reaffirmed the condition of enzootic instability (Guimarães et al., 2011). However, the herds of this breed did not have isolated outbreaks or deaths due to the disease, as observed by the owners of the herds.

The higher risk observed among males, including bulls and calves, supports previous research linking testosterone-mediated immunosuppression to increased vulnerability to protozoan infections (Kamis & Ibrahim, 1989; Prado et al., 1999; Cernetich et al., 2006). This was found in a study on *B. microti* infection in rats and was attributed to testosterone, suggesting that the male hormone is involved in reduced innate immunity in infected rats (Sasaki et al., 2013). Similarly, the highest rates of *B. bovis* infection in male cattle have been attributed to the effects of testosterone (Fereig et al., 2017). These data support the hypothesis that the higher rate of *B. bigemina* infection in Crioula Lageana males is related to testosterone and the consequent reduction in innate immunity. Further studies are required to confirm these findings.

Bulls and calves (male and female) were more likely to acquire *B. bigemina* infections than cows and heifers. Once again, sexual factors may be linked to this fact, just as age may influence infection behavior. Older animals tend to be more easily infected by the causative agents of babesiosis in cattle (Zintl et al., 2005). The literature mentions that calves have strong innate immunity against clinical disease caused by *B. bovis* (Trueman & Blight, 1978; Goff et al., 2001). Understanding how these factors converge is crucial, as incomplete passive immunity or reduced early exposure might diminish robust immune responses later in life and influence the overall dynamics of parasite transmission and disease manifestation.

None of the calves presented the clinical form of the disease in the present study. However, the chance of contracting the infection was higher in calves than in cows and heifers. It is possible that vector-resistant bovine breeds, such as the Crioula Lageana breed (Cardoso et al., 2014), may have lower rates of inoculation with the agent since the clinical disease was absent in all animal categories. This process is common in native breeds because of their natural adaptation (Solorio-Rivera & Rodríguez-Vivas, 1997). Thus, the high chance of calves acquiring hemoparasites may be due to a failure in the transfer of immunity in these animals. Once these herds are in a state of enzootic instability for *B. bigemina*, some cows that do not have constant contact with the agent do not properly transfer antibodies via colostrum to their calves.

The presence of ticks that parasitize animals is a predisposing factor for infection. Tick infestation is also a risk factor for babesiosis (Silva et al., 2014). Teleogins are the only form of tick development that can infect *B. bigemina* for later transmission of protozoans to their eggs (Riek, 1964; Mehlhorn & Shein, 1985). However, transmission to vertebrate hosts occurs through nymphs, adult females, and males (Solorio-Rivera & Rodríguez-Vivas, 1997). No accurate tick counting or developmental stage parasitization was performed on Crioula Lageana animals.

Among the infection-related factors, contact of cattle with other species (*p* = 0.037) may increase the chance of acquiring *B. bigemina* protozoan by 1.570 times. *Rhipicephalus microplus* ticks may interact with other non-ruminant species, thus introducing treatment-resistant tick populations (Solorio-Rivera & Rodríguez-Vivas, 1997). This contact becomes a source of tick infestation for cattle, thus increasing the number of ticks in the bovine population and, consequently, the chances of infection by *Babesia* spp.

Veterinary care played a role contrary to the expectations of *B. bigemina* control of babesiosis. Properties that did not have regular visits (ideally monthly) from professionals had a higher number of negative animals than those that received consultations. This is in agreement with the regular presence of veterinarians with lower seropositivity rates for Leptospira spp. in herds in Rio de Janeiro (Lilenbaum & Souza, 2003). In this study, the regular presence of a veterinarian was shown to increase the risk of infection. This may be associated with a lack of errors in applying control measures suggested by professionals, leading to inefficient disease control. However, further studies are needed to determine the reason for the association between the presence of a veterinarian and a greater chance of infection in the herd.

We concluded that Crioula Lageana cattle are in enzootic instability for *B. bigemina* according to the PCR technique. Male sex, category (bulls and calves), and tick infestation were linked to parasite acquisition. The main risk factors for infection were contact with other animal species and a lack of regular veterinary care. In integrating these considerations, the results of this study deepen our understanding of *B. bigemina* epidemiology in a valuable local breed and underscore the importance of multifaceted approaches to disease prevention and control.
